# Flow Cytometric Assessment of Pertactin- and Tetanus Toxoid-Specific B-Cell Kinetics After Tdap Booster Vaccination in Healthy Adults

**DOI:** 10.3390/vaccines14040297

**Published:** 2026-03-26

**Authors:** Mirjam J. Esser, Annieck M. Diks, Liesbeth E. M. Oosten, Rick J. Groenland, Bas de Mooij, Cristina Teodosio, Gertjan J. A. Driessen, Jacques J. M. van Dongen, Magdalena A. Berkowska

**Affiliations:** 1 Department of Pediatrics, MosaKids Children’s Hospital, Maastricht University Medical Center, P. Debyelaan 25, 6229 HX Maastricht, The Netherlands; 2 Research Institute for Oncology and Reproduction (GROW), Maastricht University, 6200 MD Maastricht, The Netherlands; 3 Department of Immunology, Leiden University Medical Center, Albinusdreef 2, 2333 ZA Leiden, The Netherlandsm.berkowska@erasmusmc.nl (M.A.B.); 4 Department of Hematology, Leiden University Medical Center, Albinusdreef 2, 2333 ZA Leiden, The Netherlands; 5 Centro de Investigación del Cáncer-Instituto de Biología Molecular y Celular del Cáncer (CIC-IBMCC, USAL-CSIC-FICUS), Department of Medicine, University of Salamanca, 37007 Salamanca, Spain; 6 Cytometry Service, NUCLEUS, University of Salamanca, 37007 Salamanca, Spain; 7 Institute of Biomedical Research of Salamanca (IBSAL), 37007 Salamanca, Spain; 8 Department of Immunology, Erasmus Medical Center, Dr. Molewaterplein 40, 3015 GD Rotterdam, The Netherlands

**Keywords:** Tdap vaccine, flow cytometry, pertactin, memory B cells, plasma cells, antibody, pertactin-specific B-cell responses

## Abstract

**Background:** Despite its high vaccination coverage, pertussis remains a public health concern due to waning vaccine-induced immunity and the emergence of pertactin (Prn)-negative strains. Nevertheless, anti-Prn antibodies and memory B cells elicited by vaccinations may contribute to long-term immunity and protection against Prn-positive strains. While most vaccination studies focus on serum antibodies, data on memory B cells remain limited. **Methods:** In this study, we implemented a flow cytometry-based approach to characterize Prn-specific B-cell fluctuations following Tdap booster vaccination in five healthy adults. Total and Prn- and tetanus toxoid fragment C (TTC)-specific plasma cells and memory B cells were analyzed at baseline and at 7, 14, 21, and 90 days post-vaccination using Prn Klickmers^®^ and TTC tetramers. Following this, cellular responses were correlated with antigen-specific serum IgG and IgA levels. **Results:** Prn-specific and TTC-specific memory B cells increased on days 14 and 7 post-vaccination, respectively, accompanied by a phenotypic shift from IgMD+ to IgG+ cells. Clear expansions of total as well as Prn- and TTC-specific plasma cells occurred on day 7. These plasma cells primarily comprised IgG+, but an increase in Prn-specific IgA+ plasma cells was also observed. The numbers of Prn-specific IgG+ memory B cells on day 7 post-vaccination correlated weakly with serum anti-Prn IgG levels at later time points. **Conclusion:** To our knowledge, this is the first study to use flow cytometry to evaluate Prn-specific B-cell responses and report their fluctuations over time following vaccination. These findings support the potential of this method to complement serological assays and improve our understanding of vaccine-induced immunity.

## 1. Introduction

Pertussis, or whooping cough, is a highly contagious respiratory disease caused by the bacterium *Bordetella (B.) pertussis*. In the pre-vaccine era in the US, it affected 872 per 100,000 people annually, and most cases occurred in children under the age of 5 [1]. Whole-cell pertussis (wP) vaccines introduced in the 1940s dramatically reduced the number of pertussis cases [2]; however, the presence of lipopolysaccharides and other bacterial components in the vaccine led to some rare but severe adverse events, prompting the development of safer and better-defined acellular pertussis (aP) subunit vaccines [3], which were introduced in the late 1990s and early 2000s [4,5]. Early aP vaccines contained either a single component, pertussis toxin (PT), or two components, PT and filamentous hemagglutinin (FHA) [6]. Additional pertussis proteins, such as pertactin (Prn) and fimbriae (FIM2, FIM3), were introduced to increase vaccine efficacy and ensure broader, longer-lasting protection. Currently, aP vaccines used in national immunization programs contain either three (PT, FHA, Prn) or five (PT, FHA, Prn, FIM2, FIM3) pertussis proteins, often in combination with diphtheria and tetanus toxoids (DTaP vaccines).

Although aP vaccines that contain more proteins seem more effective in preventing whooping cough than vaccines with fewer proteins [7], the introduction of aP vaccines coincided with an increase in the number of pertussis cases [8]. Despite widespread vaccination programs, pertussis outbreaks continue to occur every few years [9]. Several factors contribute to the difficulty in controlling pertussis, including high transmission due to asymptomatic colonization in older children and adults, declining vaccination rates, and the limited durability of antibody responses following aP vaccination [8]. In addition, aP vaccines induce a Th2-skewed T-cell response, which differs from the Th1/Th17-polarized response elicited by wP vaccines and is less effective in preventing bacterial colonization, transmission, and the development of long-term protective memory [10,11]. Furthermore, mutations in *B. pertussis* strains may provide *B. pertussis* with potential vaccine-escape mechanisms, as illustrated by the recent increase in Prn-deficient strains [12,13].

Pertussis-specific serum antibody levels, primarily anti-PT IgG, have traditionally been used to evaluate protective immunity [14,15]. In addition to the role of anti-PT, several studies suggest that anti-Prn antibodies are crucial for protection [16,17,18]. Interestingly, vaccination trials with an intranasal life-attenuated vaccine highlighted the role of anti-Prn antibodies [19,20], wherein vaccine strains failed to colonize 20% of vaccinated individuals due to the presence of serum anti-Prn antibodies [20]. Moreover, anti-Prn antibodies seem to decay relatively slowly compared with other anti-pertussis antibodies [21,22]. For example, in 11 adults recovering from pertussis, the mean anti-PT antibody titer was 11-fold higher at 2 months than at baseline, but only 2-fold higher at 28 months, whereas the mean anti-Prn antibody titer was 5-fold greater at 2 months and remained 4-fold greater at 28 months [21]. Similarly, after vaccination with the third DTaP5 dose, anti-PT IgG declined 21-fold between 1 and 23 months, while anti-Prn IgG decreased 11-fold during the same period [23]. Studies suggesting a protective role of antibodies against other *B. pertussis* vaccine proteins (FHA, FIM2, FIM3) are limited and inconclusive [24]. Despite the abovementioned observations, no single internationally accepted correlate of protection has been established, leading to ongoing efforts to identify new correlates of protection.

Memory B cells are essential for long-term immunity as they can persist for years after infection or vaccination and facilitate a rapid response upon antigen re-encounter [25,26,27]. For example, memory B cells against variola virus were detected more than 50 years after smallpox vaccination [28]. Moreover, memory B cells against SARS-CoV-2 were shown to correlate with protection against COVID-19, as individuals with higher post-vaccination memory B-cell frequencies were less likely to experience breakthrough infection [29]. Together, these findings highlight the potential value of memory B cells as more reliable indicators of durable immunity and suggest that assessing pertussis-specific memory B-cell responses alongside traditional serology may improve our understanding of long-term protection against pertussis.

Pertussis-specific memory B cells have been detected up to decades post-vaccination, even when corresponding antibody levels have been low [30]. In the first year following immunization, memory B-cell frequencies often correlate with serum antibody levels [31,32]. However, this correlation diminishes over time, as antibodies wane while memory B cells persist. The reported kinetics and magnitude of memory B-cell responses against different pertussis proteins differ between studies. While some authors have reported elevated memory B-cell numbers against all studied pertussis antigens (PT, Prn, and FHA) one month after pertussis infection and vaccination [31,33,34], others have observed increased numbers of memory B cells against FHA and Prn, but not against PT [35]. Prn- and FHA-specific memory B-cell levels were found to typically be higher than levels of memory B cells that recognize PT [34], and, in contrast to other pertussis antigen-specific B cells, remained significantly increased one year post-vaccination [31]. These findings suggest that Prn-specific memory B cells may play an important role in long-term protection against pertussis.

One of the biggest challenges in studying antigen-specific memory B cells is their low frequency in the bloodstream, which necessitates a highly sensitive approach [31]. Until recently, most studies on B cells specific to pertussis proteins utilized an Enzyme-Linked ImmunoSpot (ELISpot) assay in which peripheral blood mononuclear cells (PBMCs) or purified B cells are stimulated to induce memory B-cell differentiation into antibody-secreting cells (ASCs), which can be tested against antigens of interest [36]. Although effective, this method is highly laborious and has a limited capacity in assessing multiple B-cell types.

Advancements in high-throughput, multicolor flow cytometry have provided an alternative to ELISpot assays for studying antigen-specific immune cells. While flow cytometry is commonly used to study B cells recognizing SARS-CoV-2 or influenza viruses, and has been established for some bacterial antigens, it has not been used in the context of *B. pertussis* [37,38,39,40].

In this exploratory study, we developed a flow cytometry-based approach to investigate Prn-specific B cells in adults following booster Tdap vaccination. This high-throughput approach can be applied to larger cohorts to improve our understanding of the role of Prn in protective immunity against pertussis.

## 2. Materials and Methods

### 2.1. Study Design and Sample Collection

This exploratory study was performed at the Department of Immunology of the Leiden University Medical Center (LUMC; Leiden, The Netherlands), approved by the Medical Ethics Committee of Leiden/The Hague/Delft (NL57973.058.16, 11 January 2017), and registered in the EU Clinical trial registry (EudraCT 2016-002011-18). This study was conducted in accordance with the Declaration of Helsinki. Written informed consent was obtained before enrolment, and the inclusion and exclusion criteria were the same as those published in [41]. In short, adults who were (1) healthy, as evaluated by a questionnaire; (2) had blood hemoglobin levels within the normal range; (3) had no suspected exposure to *B. pertussis* in the past; and (4) had completed a full vaccination course according to the Dutch National Immunization Program [42] were eligible. In March and April 2022, five participants were included (4 males, 1 female; age range 31–57 years). After initial blood collection on day 0, participants were vaccinated intramuscularly with the Boostrix^®^ vaccine ((GlaxoSmithKline, London, United Kingdom) (≥2 IU/mL diphtheria toxoid (DT); ≥2 IU/mL tetanus toxoid (TT); 8 µg pertussis toxoid (PT); 8 µg filamentous hemagglutinin (FHA); and 2.5 µg pertactin (Prn), with aluminum hydroxide as the adjuvant)) [43]. Peripheral blood samples were collected in K2EDTA blood collection tubes and in serum collection tubes at baseline, and on days 7, 14, 21, and 90 after vaccination. One of the participants reported having a common cold on day 21.

### 2.2. Evaluation of Vaccine-Specific Immunoglobulin Levels in Serum

Serum levels of IgA and IgG against the vaccine components (PT, FHA, Prn, DT, and TT) and against fimbriae type 2 and 3 (FIM2/3) were determined by bead-based multiplex immunoassay at the Dutch National Institute for Public Health and the Environment (RIVM) [44].

### 2.3. Flow Cytometric Analysis of Circulating B-Cell Subsets

B-cell staining was performed on freshly collected whole blood, and samples were processed according to the EuroFlow bulk lysis protocol [45]. In total, 1.5–2 mL of blood was incubated for 15 min with lysis solution (NH_4_Cl), and cells were pelleted and washed twice with wash buffer (PBS/0.2% BSA/0.1% NaN_3_). Cells were counted and resuspended in wash buffer to a total of 1 × 10^7^ cells in 40 µL.

Tetanus toxin C (TTC)-fragment tetramers, conjugated to APC and PE fluorochromes, were provided by the Department of Rheumatology (LUMC, The Netherlands) and prepared as described in [46]. A His-tagged Prn fragment encompassing Prn residues 35–610 (Prn58-His) from the Tohama I strain was expressed in the *E. coli* BL21(DE3) strain from a codon-optimized gene cloned into a pET28-derived vector. Protein was extracted from the insoluble fraction using PBS with 4 M urea, purified using an AKTA instrument (Miami, FL, USA), and refolded using PBS with 0.5 M urea. Protein purity was higher than 95%, with no signs of protein degradation. A single batch of Prn58-His (930 µg at 930 µg/mL) was biotinylated in a single chemical biotinylation reaction via NHS-ester activation to covalently couple biotin to solvent-exposed primary amines at a fixed ratio of 100 μg biotin per of protein. Then, biotinylated protein was assembled with Klickmer^®^ fluorescent dextrans (Immudex, Philadelphia, PA, USA) in both phycoerythrin (PE) and allophycocyanin (APC) formats using a ligand-to-dextramer ratio of 10:1, strictly following the manufacturer’s protocol to ensure optimal multivalent loading. All final reagents were titrated and standardized to a working concentration of 0.186 µg of protein per test.

Cells were stained with a panel of antibodies to discriminate major B-cell populations and antigen-specific B cells using Prn Klickmer^®^ or TTC tetramers (Supplementary Table S1A). The antibody panel was simplified from the validated and previously published EuroFlow IgH isotype B-cell (BIgH) panel [47,48,49]. The primary modification was the replacement of IgG1-4 and IgA1-2 subclasses with antibodies that detect IgG and IgA, allowing for the inclusion of antigen Klickmers^®^ and tetramers. Next, cells were intracellularly stained using a Fix&Perm kit (Nordic MUbio, Susteren, The Netherlands) according to the manufacturer’s protocol (Supplementary Table S1A). They were then incubated, washed, and directly analyzed using a three-laser Aurora (Cytek, Fremon, CA, USA) at the Flow Cytometry Core Facility of the LUMC. A median of 4–7 × 10^6^ events were acquired per participant per time point, with a median of 115,512 (IQR 92,146–157,230) B cells.

To calculate absolute cell numbers, Perfect-Count Microspheres^TM^ (Cytognos, Salamanca, Spain) were used as previously described in [50]. In short, exactly 50 µL of peripheral blood was mixed with antibodies according to the Perfect-Count panel (Supplementary Table S1B) and incubated for 30 min. Lysis solution was added, and the cells were incubated for an additional 10 min. Then, 50 µL of Perfect-Count Microspheres^TM^ was added, and samples were immediately acquired using an FACSCanto II (Becton Dickinson Biosciences, San Jose, CA, USA).

### 2.4. Data Analysis and Statistics

Flow cytometry data were analyzed with Infinicyt software (version 2.0, Cytognos) according to the gating strategy described in Supplementary Table S2, which was adapted from the previously published gating strategy for the EuroFlow B-cell tube [49]. B cells were defined as TTC-specific if they were double-positive for TTC-APC and TTC-PE, and as Prn-specific if they were double-positive for Prn-APC and Prn-PE. No T-cell binding to TTC and Prn was observed. Cell populations were subdivided only when the parent population contained a minimum of 30 events. For visualization of the data, GraphPad Prism version 10.0 was used, and for statistical analysis, SPSS version 22.0 was used. To test longitudinal changes in median cell numbers and antibody levels compared with baseline, Wilcoxon signed-rank tests for paired samples were used, while correlations between cell numbers and antibody levels were evaluated using Spearman’s Rank Correlation, with a *p*-value < 0.05 considered statistically significant. Because of the explorative nature of the study, no multiple testing correction was performed.

## 3. Results

### 3.1. Serological Response to Vaccination

**Figure 1:**
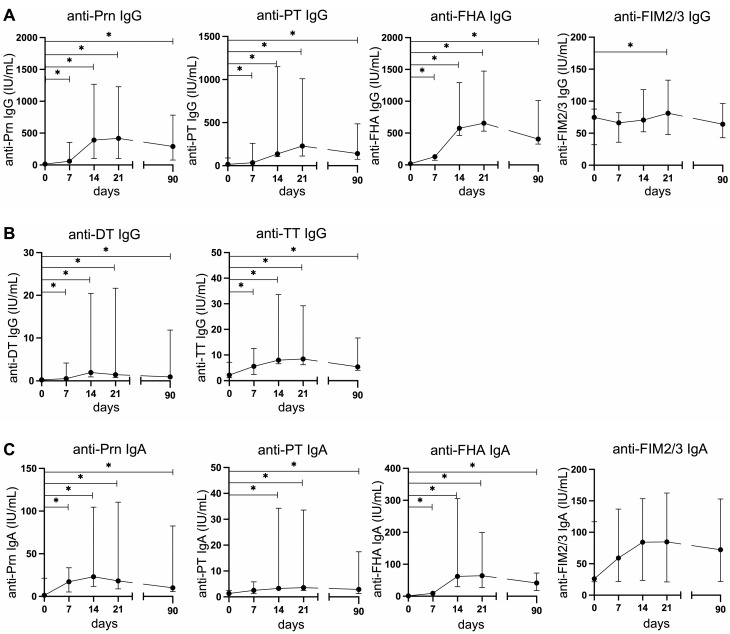
Serum vaccine-specific IgG and IgA antibody levels post-vaccination. (**A**) IgG levels against Prn, PT, FHA, and FIM2/3, (**B**) IgG levels against DT and TT, and (**C**) IgA levels against Prn, PT, FHA, and FIM2/3. Median values were calculated to construct the plots. Wilcoxon matched-pair signed-rank tests were used to assess longitudinal differences. * *p* < 0.05.

All participants responded to vaccination with an increase in antibody levels against vaccine components (Figure 1). IgG levels against all vaccine antigens were statistically significantly increased from day 7 onwards, while IgG levels against FIM2/3, which is absent in the vaccine, were increased on day 21 only (Figure 1A,B). There was a statistically significant increase in IgA levels against PT and Prn from day 7 onwards, and against PT from day 14 onwards (Figure 1C). There was no significant increase in IgA levels against FIM2/3. The peak antibody responses against vaccine components were observed on day 14 or 21 post-vaccination, and by day 21, all participants had TT-IgG concentrations above the protective cut-off value of 0.1 IU/mL.

### 3.2. Strong Increase in IgG+ Total Plasma Cells on Day 7 Post-Vaccination

**Figure 2:**
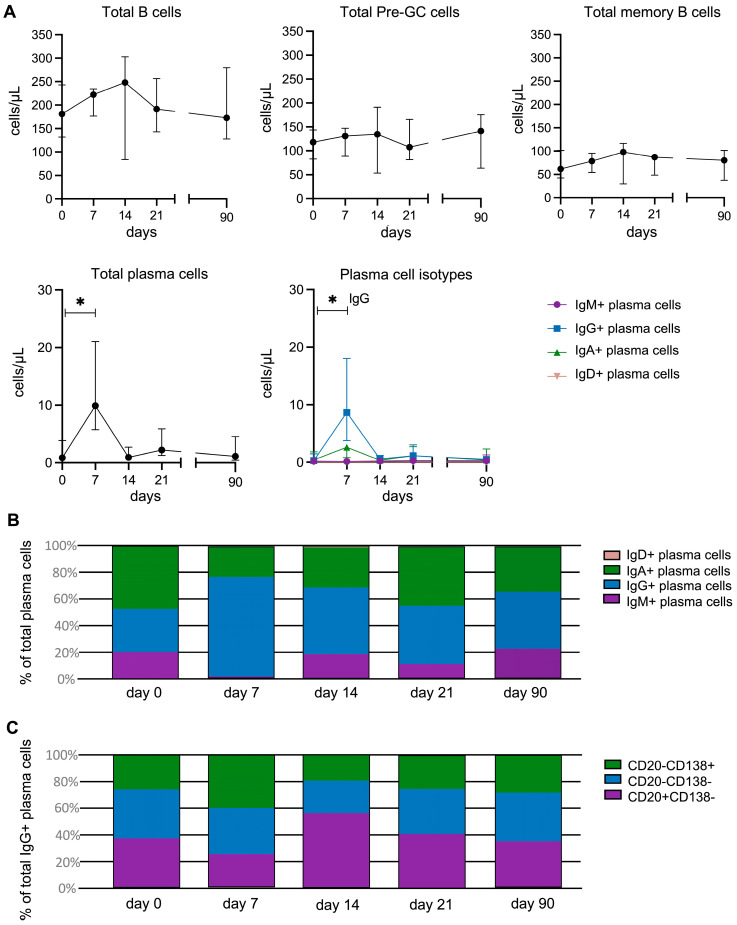
Kinetics of B-cell and plasma-cell subsets over time following vaccination. (**A**) Changes in numbers of B cells, pre-GC B cells, memory B cells, plasma cells, and plasma-cell isotypes. Each point represents a median value with the interquartile range calculated for 5 participants. The black line connects the median values at the different time points. The break in the line indicates the interval between 21 and 90 days. The Wilcoxon matched-pairs signed-rank test was used to assess longitudinal differences within each cell subset. * *p* < 0.05 (**B**) Distribution of plasma-cell isotypes over time. (**C**) Distribution of maturation stages within total IgG+ plasma cells over time. Maturation stages were based on CD20 and CD138 expression.

At baseline, all participants had normal white blood cell (4.2–7 × 10^6^ cells/mL; normal range 4–10 × 10^6^ cells/mL) and lymphocyte counts (1.5–2.7 × 10^6^ cells/mL; normal range 1–3.5 × 10^6^ cells/mL). No statistically significant changes were observed in total B-cell, pre-germinal center (pre-GC) B-cell, or memory B-cell numbers on days 7, 14, 21, and 90 post-vaccination compared with baseline (Figure 2A). In contrast, total plasma cells showed pronounced expansion on day 7 following vaccination compared with baseline (median 9.91 cells/µL (IQR, 5.7–21.1) vs. 0.85 cells/µL (0.56–3.86), range of 2.0–49.7-fold increase, *p* = 0.043), returning to baseline levels by day 14. This increase was the most prominent for IgG+ plasma cells (median 8.65 cells/µL (IQR, 3.76–18.05) vs. 0.29 cells/µL (0.20–1.47) at baseline, *p* = 0.043), followed by a statistically non-significant increase in IgA+ plasma cells (Figure 2A). Consequently, the distribution of plasma-cell subsets on day 7 was skewed towards IgG+ cells (76% IgG+ plasma cells, 23% IgA+ plasma cells) compared with baseline (33% IgG+ vs. 47% IgA+ plasma cells, Figure 2B). The contribution of IgG+ plasma cells to total plasma cells decreased after day 7, but they remained the most abundant plasma cell subset on day 90 (43% IgG+ vs. 33% IgA+ plasma cells).

Plasma-cell maturation stages were determined based on the expression of CD20 and CD138. At baseline, 37% of IgG+ plasma cells had the least mature phenotype (CD20+CD138−-); these types of cells are also referred to as plasmablasts in the literature. An additional 37% showed an intermediate-maturity phenotype (CD20−CD138−), and almost 26% represented the most mature phenotype (CD20−CD138+) (Figure 2C). By day 7 post-vaccination, the absolute count of the least mature CD20+CD138− cells had increased 13.1-fold, that of the intermediate-maturity CD20−CD138− cells had increased 18.5-fold, and that of the most mature CD20−CD138+ cells had increased 30.3-fold. At this time, mature IgG+ plasma cells (CD20−CD138+) were the most abundant population, constituting 40% of total plasma cells. From day 14 onwards, the distribution of plasma cell maturation stages returned to baseline, with most cells comprising the least mature or intermediate-maturity subsets. This change was not observed for the IgM+ and IgA+ plasma cells, in which the lowest- or intermediate-maturity subsets remained the most abundant at all time points. Thus, consistent with previous findings, the expansion and maturation of predominantly IgG+ plasma cells occurred on day 7 post-vaccination [41,51].

### 3.3. Strong Increase in IgG+ Prn-Specific Plasma Cells

**Figure 3:**
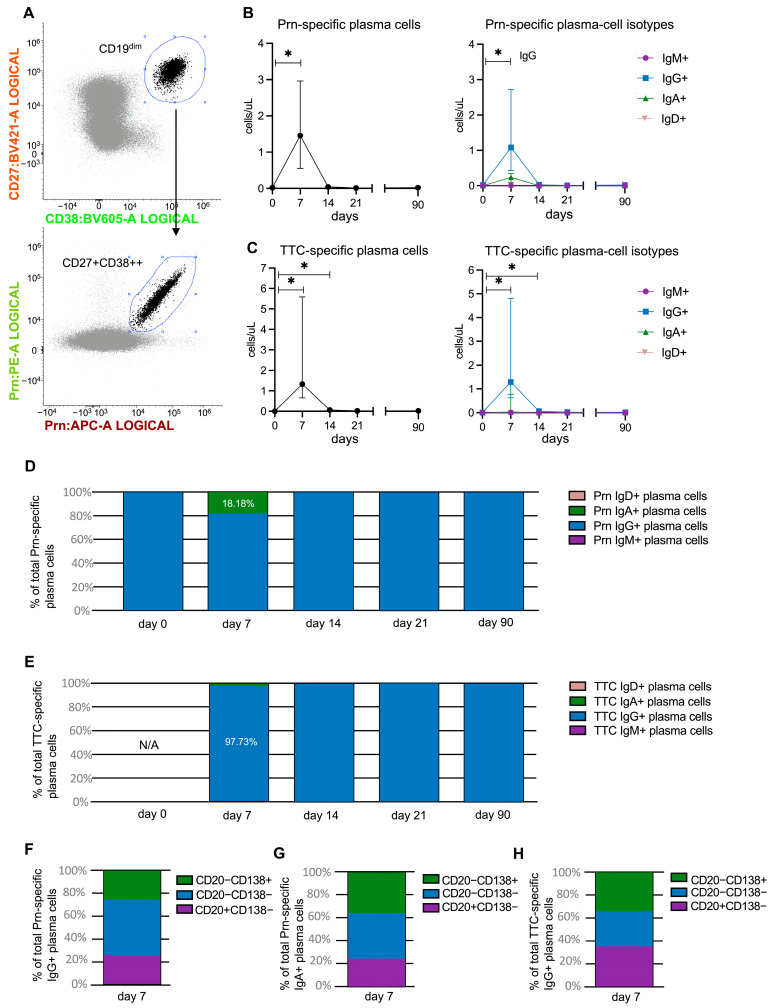
Changes in Prn-specific and TTC-specific plasma-cell subsets over time following vaccination. (**A**) Identification of CD19^dim^CD27^+^CD38^++^ plasma cells double-positive for Prn-conjugated Klickmer^®^ on day 7. Changes in the number of (**B**) Prn-specific and (**C**) TTC-specific plasma cells and plasma-cell isotypes. In (**B**,**C**), each point represents a median value with an interquartile range. The black line connects the median values at the different time points. The break in the line indicates the interval between 21 and 90 days. The Wilcoxon matched-pairs signed-rank test was used to assess longitudinal differences within each cell subset. * *p* < 0.05 (**D**) Distribution of plasma-cell isotypes over time within the (**D**) Prn-specific and (**E**) TTC-specific plasma cells. (**F**–**H**) Distribution of the different maturation stages within Prn-specific IgG+ plasma cells, Prn-specific IgA+ plasma cells, and TTC-specific IgG+ plasma cells on day 7 post-vaccination. Maturation stages were based on CD20 and CD138 expression.

Compared with the total plasma-cell population, Prn-specific plasma cells showed even stronger expansion on day 7 (Figure 3A,B). Their numbers increased from a median of 0.02 (IQR, 0.01–0.05) cells/µL at baseline to 1.46 (0.56–2.97) cells/µL on day 7, reflecting a 4–442-fold increase (*p* = 0.043). Most Prn-specific plasma cells were IgG+, with a median of 1.08 (IQR, 0.43–2.72) cells/µL on day 7. Additionally, Prn-specific IgA+ plasma cells, which were undetectable at baseline, increased to 0.24 (IQR, 0.09–0.35) cells/µL on day 7. Consequently, on day 7, 18% of Prn-specific plasma cells were IgA+, while at all other time points, all detected Prn-specific plasma cells were IgG+ (Figure 3D). TTC-specific plasma cells, which were used as a control for the vaccination response, also rose from undetectable levels at baseline to 1.33 (IQR, 0.66–5.59) cells/µL on day 7 (*p* = 0.043) (Figure 3C). They remained significantly elevated on day 14 (median 0.06 (IQR, 0.04–0.10) cells/µL, *p* = 0.042) before returning to baseline levels by day 21. Similarly to Prn-specific cells, IgG+ plasma cells were the most expanded isotype within the TTC-specific population and comprised 98% of the TTC-specific plasma cells on day 7. The increase in IgA+ plasma cells was negligible, representing only 2% of TTC-specific plasma cells at this time point (Figure 3E).

On day 7, Prn- and TTC-specific plasma cells accounted for 15% and 13% of the total circulating plasma-cell pool, respectively. Since their frequencies were low at other time points, the maturation stages of Prn- and TTC-specific plasma cells were analyzed only on day 7. Within the Prn-specific IgG+ and IgA+ plasma cells, those with an intermediate phenotype were the most abundant (48% and 40%, respectively) (Figure 3F,G). In contrast, within the TTC-specific IgG+ plasma cells, those with an immature phenotype were the most abundant (35%) (Figure 3H). This differed from the total IgG+ plasma-cell pool, where mature plasma cells were the most abundant subset on day 7 (Figure 2C), suggesting that Prn- and TTC-specific plasma cells might have somewhat different maturation kinetics compared with the total plasma-cell pool, which likely consists predominantly of cells targeting other Tdap vaccine antigens.

### 3.4. Longitudinal Changes in Prn-Specific Memory B-Cell Subsets Following Vaccination

**Figure 4:**
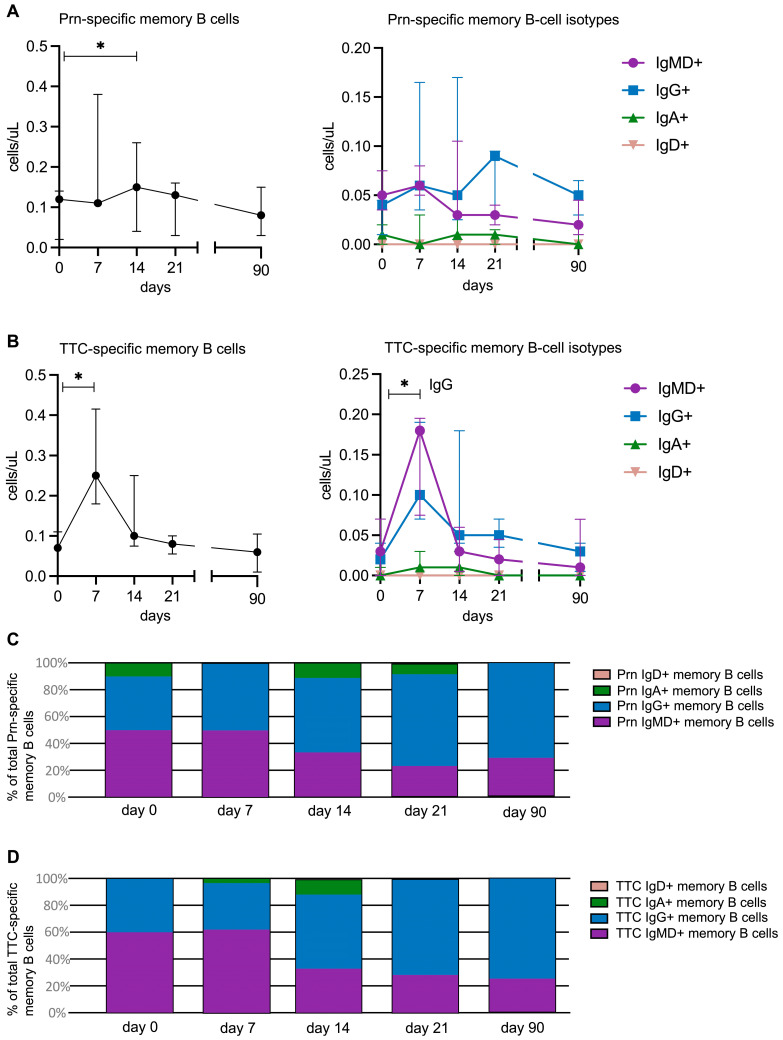
Changes in Prn- and TTC- specific memory B-cell subsets over time following vaccination. Changes in the numbers of (**A**) Prn- and (**B**) TTC-specific memory B cells and B-cell subsets over time. In (**A**,**B**), each point represents a median value with an interquartile range. The black line connects the median values at the different time points. The break in the line indicates the interval between 21 and 90 days. The Wilcoxon matched-pairs signed-rank test was used to assess longitudinal differences within each cell subset. * *p* < 0.05. (**C**) Distribution of memory B-cell isotypes over time within the Prn-specific memory B cells. (**D**) distribution of memory B-cell subsets over time within the TTC-specific memory B cells. Median values were calculated to construct the graphs.

Prn-specific and TTC-specific B cells were identified as CD19+CD45+CD20+ and double-positive for Prn Klickmer^®^ or TTC tetramer (Supplementary Table S2). For all participants and time points combined, a median of 75 (IQR, 46–103) Prn-specific memory B cells and 70 (IQR, 37–106) TTC-specific memory B cells were measured. Prn-specific memory B-cell numbers showed a small but statistically significant increase on day 14 compared with baseline (median 0.15 (IQR, 0.07–0.26) cells/µL vs. 0.12 (0.05–0.14) cells/uL, *p* = 0.039) (Figure 4A). These cells represented 0.15% of total circulating memory B cells on day 14 and 0.19% at baseline. The Prn-specific memory B-cell numbers returned to baseline levels by day 21 and remained stable until day 90. Within the Prn-specific memory B-cell subsets, IgG+ cells showed the most pronounced increase on day 21 (median 0.09 (IQR, 0.02–0.10) cells/µL vs. 0.04 (0.01–0.05) cells/µL at baseline, *p* = 0.104) (Figure 4A). However, this increase was not statistically significant. At that time, all Prn-specific IgG+ memory B cells were CD27+. No significant fluctuations were observed over time in IgMD+, IgA+, and IgD+ Prn-specific memory B cells (Figure 4A). TTC-specific memory B cells showed a statistically significant increase on day 7 compared with baseline (*p* = 0.043) and returned to baseline levels by day 21 (Figure 4B). This increase was most pronounced in the IgMD+ (*p* = 0.066) and IgG+ subsets (*p* = 0.043).

At baseline and day 7, the IgMD+ subset was the predominant memory B-cell subset within both the Prn-specific population (50% at both day 0 and 7) and TTC-specific population (60% on day 0 and 62%, on day 7) (Figure 4C,D). However, by day 14, a shift occurred, with IgG+ memory B cells becoming the most abundant subset, accounting for 56% of memory B cells within both the Prn- and TTC-specific populations. This dominance of IgG+ memory B cells persisted through day 90 (Figure 4A,B).

### 3.5. Correlation Between Vaccine-Specific Antibody Levels and Prn-Specific Plasma Cells or Memory B Cells

To assess the extent to which elevated antigen-specific plasma-cell numbers predict an increase in antibody levels, we evaluated the correlation between Prn- and TTC-specific plasma-cell numbers on day 7 and serum levels of anti-Prn and anti-TT IgG antibodies on days 14, 21, and 90 post-vaccination. Although there was a trend toward higher anti-Prn IgG levels in participants with higher Prn-specific IgG+ plasma-cell numbers (correlation coefficient 0.700, *p* = 0.188 at all time points), no statistically significant correlations were observed (Supplementary Figure S1). Furthermore, no significant correlations were observed between TTC-specific IgG+ plasma-cell numbers on day 7 and serum levels of anti-TT IgG at any time point (Supplementary Figure S1).

Additionally, we evaluated whether Prn- and TTC-specific memory B-cell numbers on days 7, 14, and 21 correlated with corresponding serum IgG levels on days 7, 14, 21, and 90 post-vaccination. Prn-specific IgG+ memory B cells on day 7 were positively correlated with anti-Prn IgG levels at all measured post-vaccination time points (all *p* = 0.037) (Supplementary Figure S2A). No significant correlations were detected between Prn-specific IgA+ memory B-cell numbers and anti-Prn IgA levels or between TTC-specific IgG+ memory B-cell numbers and anti-TT IgG levels (Supplementary Figures S2 and S3). Together, these results indicate that the post-vaccination expansion of Prn-specific memory B cells is associated with higher anti-Prn IgG concentrations. Although a similar trend was observed for Prn-specific plasma cells, the association did not reach statistical significance, likely due to the limited sample size. This indicates that flow cytometric detection of antigen-specific memory B cells may serve as an additional surrogate marker of vaccine response.

## 4. Discussion

Pertactin, alongside PT and FHA, is a key component of most acellular pertussis vaccines, and several studies suggest that responses against Prn may correlate with protection from whooping cough. While Prn-specific antibodies are commonly assessed in clinical trials, assessment of Prn-specific memory B cells is usually restricted to research settings and based on the reliable but laborious ELISpot assay. In this explorative study, we set up a flow cytometry-based assay to study Prn-specific B cells in five healthy adults following Tdap booster vaccination. Additionally, we studied TT- and TTC-specific antibodies and B cells as a control, representing a hallmark of the tetanus toxoid vaccination response. Prn- and TTC-specific plasma-cell numbers showed a pronounced increase on day 7 post-vaccination, followed by a rise in corresponding antibody levels on day 14. TTC- and Prn-specific memory B cells increased on days 7 and 14, respectively, with a clear transition from a predominantly IgMD+ phenotype to an IgG+ phenotype from day 14 onwards. Prn-specific memory B cells on day 7 post-vaccination correlated weakly with anti-Prn IgG antibody levels at all subsequent time points. Collectively, these exploratory findings indicate that flow cytometric assessment of Prn-specific B-cell subsets is feasible and, when combined with serology, provides complementary insight into long-term pertussis immunity.

Four out of five participants presented with an increase in Prn-specific IgA+ plasma cells and antibodies on day 7 post-vaccination, but not in TTC-specific IgA+ plasma cells. While IgA responses are induced by mucosal exposure to a pathogen, they are usually uncommon following an intramuscular vaccination. An increase in Prn-specific IgA+ plasma cells and antibodies may reflect prior exposure to circulating *B. pertussis* [52,53]. Although studies comparing serum IgA response following intramuscular vaccination between previously infected and naive individuals are scarce [53], animal and human studies on other respiratory pathogens suggest that prior mucosal exposure can enhance subsequent systemic IgA responses [54,55]. Since mucosal IgA is important in preventing pathogen colonization and transmission [56], an increase in IgA following vaccination will likely be beneficial. However, to our knowledge, the relationship between systemic and mucosal IgA in pertussis immunity has not yet been directly investigated. In one study, mucosal anti-Prn IgG antibodies, but not IgA, increased following intramuscular Tdap-IPV vaccination [57]. In a SARS-CoV-2 study, where most participants had experienced at least one prior infection, post-vaccination spike-specific IgA levels in serum correlated with those in saliva [58]. Future studies that include sampling of respiratory secretions will provide additional insight beyond the systemic response alone.

Investigating the cellular immune response against pertussis provides additional insight into immune memory in the context of waning antibody levels. Long-term immune protection is primarily mediated by long-lived plasma cells and memory B cells, which rapidly proliferate, undergo class switching, and differentiate into antibody-secreting plasma cells upon antigen re-encounter [59]. Prior studies have shown that total memory B-cell numbers remain relatively stable following vaccination [50], likely due to low frequencies of antigen (Ag)-specific memory B cells in the peripheral blood. For example, after an aP booster vaccination in 9-year-old children, only 0.1–0.4% of the memory B cells were vaccine antigen-specific [31]. This indicates that changes within antigen-specific memory subsets are difficult to detect when assessing the total memory pool, which is consistent with our findings. In contrast, we observed a transient increase in Prn-specific memory B cells on day 14 and in TTC-specific memory B cells on day 7 post-vaccination. These results reinforce the value of an Ag-specific approach to capturing fluctuations in memory B-cell populations after vaccination or infection.

Remarkably, the peak in Prn- and TTC-specific memory B cells in our study occurred earlier than in previous studies that collected samples around day 28 post-vaccination. Several reports observe increases in memory B cells on day 28 post-vaccination [31,35], whereas in our cohort, antigen-specific memory B-cell levels had nearly returned to baseline by day 21. Consistent with an early increase, in some studies, pathogen-specific B cells reached their peak at 2 weeks after booster vaccination, which aligns more closely with our findings [32,60]. In addition, following SARS-CoV-2 infection, spike-specific memory B cells have been detected from day 10-14 in some individuals [61]. These findings suggest that memory B-cell responses may arise early after infection or vaccination, especially after re-exposure, and that early sampling post-vaccination may be needed to accurately capture the peak memory B-cell response. Studies only initiating measurements from one month post-vaccination might miss the earlier rise in memory B-cell levels.

Previous studies reporting PT- and Prn-specific memory B cells up to a few years post-vaccination were based on ELISpot assays [32,62]. Comparable flow cytometry studies assessing pertussis-specific B cells are currently lacking, which limits direct comparison with our findings. In contrast to the ELISpot assay, which requires in vitro stimulation and clonal expansion, our flow cytometry approach detects Ag-specific B cells directly ex vivo without prior stimulation and therefore reflects the in vivo frequency more directly. The overall detection sensitivity of both methods seems comparable. Typically, ELISpot studies use 200,000 purified B cells per well, which results in detection of up to 100 PT- and Prn-specific memory B cells per 100,000 total B cells post-vaccination [30,32]. In our approach, processing 10 million PBMCs allowed for acquisition of a median of 115,512 (IQR 92,146–157,230) B cells per time point, of which 75 (46–103) were Prn-specific. However, in donors in whom a low number of Ag-specific B cells is expected (i.e., immunocompromised donors without a recent booster vaccination), it may be beneficial to analyze more than 10 million PBMCs or to perform B-cell enrichment prior to staining [63].

Prn is a surface protein involved in host cell adhesion, and previous studies have reported long-term Prn-specific memory B-cell immunity [31,34,35]. However, recently, Prn-deficient *B. pertussis* strains have appeared, and their frequency is increasing worldwide [12,13]. Studies in aP-vaccinated mice showed that Prn-deficient strains are more successful in colonizing the respiratory tract than Prn-positive strains as they are not neutralized by anti-Prn antibodies [64,65]. The emergence of Prn-deficient rather than, for example, PT-deficient strains, might reflect the functional redundancy of Prn for *B. pertussis* and the relatively long persistence of anti-Prn antibodies [12]. This phenomenon of vaccine-driven antigen loss has also been observed for other subunit vaccines, such as pneumococcal conjugate vaccines [66]. Our current study, which demonstrates early Ag-specific memory B-cell fluctuations via flow cytometry, was not designed to assess clinical protection. Consequently, longitudinal follow-up to assess B-cell immunity, together with vaccine-effectiveness data, is required to decide whether Prn should be replaced or supplemented in future aP formulations.

This study has several limitations. First, it was an exploratory study with a small number of participants, so variation in even one individual could affect the observed numbers. In addition, the low sample size limits the study’s statistical power and makes it difficult to generalize the findings to a larger population. Moreover, unrecognized subclinical infections may have contributed to inter-individual variability in antigen-specific B-cell responses. In addition, due to low cell numbers at several time points, we were not able to assess all outcomes over time. Finally, we assessed immune responses only up to three months post-vaccination, a timeframe in which increases in antigen-specific memory B cells following booster vaccination are typically observed. However, we may have missed later fluctuations in memory B-cell frequencies, as reported in other vaccination studies [67]. Therefore, the findings of this study should be interpreted with caution. Despite these limitations, this study provides novel insights and a proof of concept. To our knowledge, it is the first study to evaluate pertussis-specific plasma-cell and memory B-cell responses using flow cytometry without any purification steps and without ex vivo activation and culture steps. The flow cytometric assay is easy to perform within 3 to 4 h after the blood sample has arrived in the laboratory. This method allows for more detailed analysis of cell phenotypes and can enhance earlier findings from ELISpot studies. Our results provide a basis for future studies with larger cohorts to confirm and further explore these observations.

## 5. Conclusions

Cellular studies on pertussis-specific B-cell responses provide additional insight into underlying immune mechanisms in the context of waning humoral immunity. We observed pronounced expansion of Prn- and TTC-specific plasma cells, as well as a transient increase in Prn- and TTC-specific memory B cells. The increase in Prn-specific IgG+ memory B cells on day 7 post-vaccination correlated weakly with the increase in anti-Prn IgG at all subsequent time points. To our knowledge, this is the first study to use flow cytometry to study pertussis-specific B-cell responses. These findings highlight the potential of this method to enhance serological assays and T-cell immunity studies and to deepen our understanding of pertussis-related immunity, although confirmation in larger cohorts will be required.

## Data Availability

Requests to access the data should be directed to the corresponding author.

## Supplementary Materials

The following supporting information can be downloaded at: https://www.mdpi.com/article/10.3390/vaccines14040297/s1, Table S1A: Composition of the antigen-specific B-cell panel; Table S1B: Composition of the Perfect-Count panel; Table S2: Gating strategy; Figure S1: Correlation between vaccine-specific plasma-cell levels on day 7 as measured by flow cytometry, and vaccine-specific serum immunoglobulin levels at different time points post-vaccination. Figure S2. Correlation between Prn-specific memory B-cell levels on days 7, 14, and 21 as measured by flow cytometry, and vaccine-specific serum immunoglobulin levels at different time points post-vaccination. Figure S3. Correlation between TTC-specific memory B-cell levels on days 7, 14, and 21 as measured by flow cytometry, and vaccine-specific serum immunoglobulin levels at different time points post-vaccination.

## Abbreviations

The following abbreviations are used in this manuscript:

Prn	Pertactin
TTC	Tetanus toxoid fragment C
*B. pertussis*	*Bordetella pertussis*
wP	Whole-cell pertussis
aP	Acellular pertussis
PT	Pertussis toxin
FHA	Filamentous hemagglutinin
FIM2/3	Fimbriae type 2 and 3
DT	Diphtheria toxoid
TT	Tetanus toxoid
ELISpot	Enzyme-Linked ImmunoSpot
PBMC	Peripheral blood mononuclear cell
ASC	Antibody-secreting cell
Ag specific	Antigen-specific
