# Supplementary material for: Flow Cytometric Assessment of Pertactin- and Tetanus Toxoid-Specific B-Cell Kinetics After Tdap Booster Vaccination in Healthy Adults
Source: Vaccines (Basel). 2026 Mar 26;14(4):297. doi: 10.3390/vaccines14040297 (PMC13120655; doi:10.3390/vaccines14040297)
Supplement: Supplementary file 1 [file vaccines-14-00297-s001.zip › vaccines-4179852-supplementary.pdf]

Supplementary material

Table S1A. Composition of the antigen-specific B-cell panel.

	BV421	BV510	BV605	BV650	BV786		PerCP-Cy5.5	PerCP Vio700	PE-CF94	PE-Cy5	PE-Cy7		AF700
**Antibody**	**CD27**	**IgM**	**CD38**	**CD45**	**IgD**	**IgG**	**IgD**	**IgA**	**CD20**	**CD24**	**CD5**	**CD138**	**CD19**
**Volume (µl)**	2	2	2	20	0.5	5	2	2.5	5	10	6	5	10
**Company**	BD	Biolegend	Biolegend	BD	BD	BD	Biolegend	Miltenyi	BD	Biolegend	BD	Biolegend	BC
**Clone**	M-T271	MHM-88	HIT2	HI30	IA6-2	G18-145	IA6-2	IS11-8E	2H7	ML5	L17F12	MI15	J3-119
**Catalogue no**	562513	314522	303532	563717	740997	564230	348208	130-113-478	562295	311148	348810	356514	B76283
**Intracellular stain**	no	yes	no	no	yes	yes	yes	yes	no	no	no	no	no

PE	APC
**Prn**	**Prn**
2µl	2µl
no	no

PE	APC
**TTC**	**TTC**
0.4µl	1µl
no	no

Table S1B. Composition of the perfect count panel.

	OC515	PE		APC	PE-Cy7	PerCP-Cy5.5
**Antibody**	**CD45**	**CD16**	**CD56**	**CD3**	**CD19**	**CD36**
**Volume (µl)**	5	5	5	**2.5**	**5**	10
**Company**	Cytognos	BD	Cytognos	BD	Beckman Coulter	Immunostep
**Clone**	GA90	3G8	C5.9	SK7	J3-119	CLB-IVC7
**Catalogue no**	CYT-45OC	555407	CYT-56PE	345767	IM3628	36PP5.52-100

Table S2. Gating strategy

#1. Identification of total plasma cells	CD45+CD19^{dim}CD38++CD27+CD24–-
#2. Identification of double positive antigen-specific plasma cells	• In the ‘Prn’ tube, Prn-APC+ Prn-PE+ cells were assigned to Prn-specific plasma cells<br>• In the ‘TTC’ tube, TTC-APC+ TTC-PE+ cells were assigned to TTC-specific plasma cells<br>• The remaining plasma cells were assigned to ‘other plasma cells’
The subsequent steps were performed for each population defined in step #2	
#3. Classification of plasma cells based on isotype	• IgA+, no expression of other isotype Igs<br>• IgM+, no expression of other isotype Igs<br>• IgD double positive, no expression of other isotype Igs<br>• IgG+, no expression of other isotype Igs
#4. Definition of maturation stage	• Least mature plasma cells: CD20+CD138–-<br>• Intermediate mature plasma cells: CD20–-CD138–-<br>• Mature plasma cells: CD20–-138+
#5. Identification of total B cells	CD45+CD19+CD20+ with low side scatter characteristics
#6. Identification of double positive antigen-specific B cells	• In the ‘Prn’ tube, Prn-APC+ Prn-PE+ cells were assigned to Prn-specific B cells<br>• In the ‘TTC’ tube, TTC-APC+ TTC-PE+ cells were assigned to TTC-specific B cells<br>• The remaining plasma cells were assigned to ‘B cells’
The subsequent steps were performed for each population defined in step #6	
#7. Identification of class-switched memory B-cell subsets based on isotype.	• IgA+CD27^{het}, no expression of other isotype Igs<br>• IgG+CD27^{het}, no expression of other isotype Igs<br>• IgD double positive CD27+
#8. Identification of non-switched memory B cells and pre-GC B cells	• CD27+IgM+ were assigned to IgMD+ memory B cells<br>• CD27–-IgM+ were assigned to pre-GC B cells
#9. Subclassification of class-switched memory B cells based on CD27 expression	• CD27+<br>• CD27–-
#10. Subclassification of pre-GC B cells	• Immature pre-GC B cells: CD38+CD24+<br>• Naive CD5+ B cells: CD38–-CD24dimCD5+<br>• Naive CD5- B cells: CD38–-CD24dimCD5–-

Phenotypic descriptions used to define B-cell subsets by manual analysis within Infinicyt software. The removal of debris and doublets is not indicated in the analysis but was also performed.

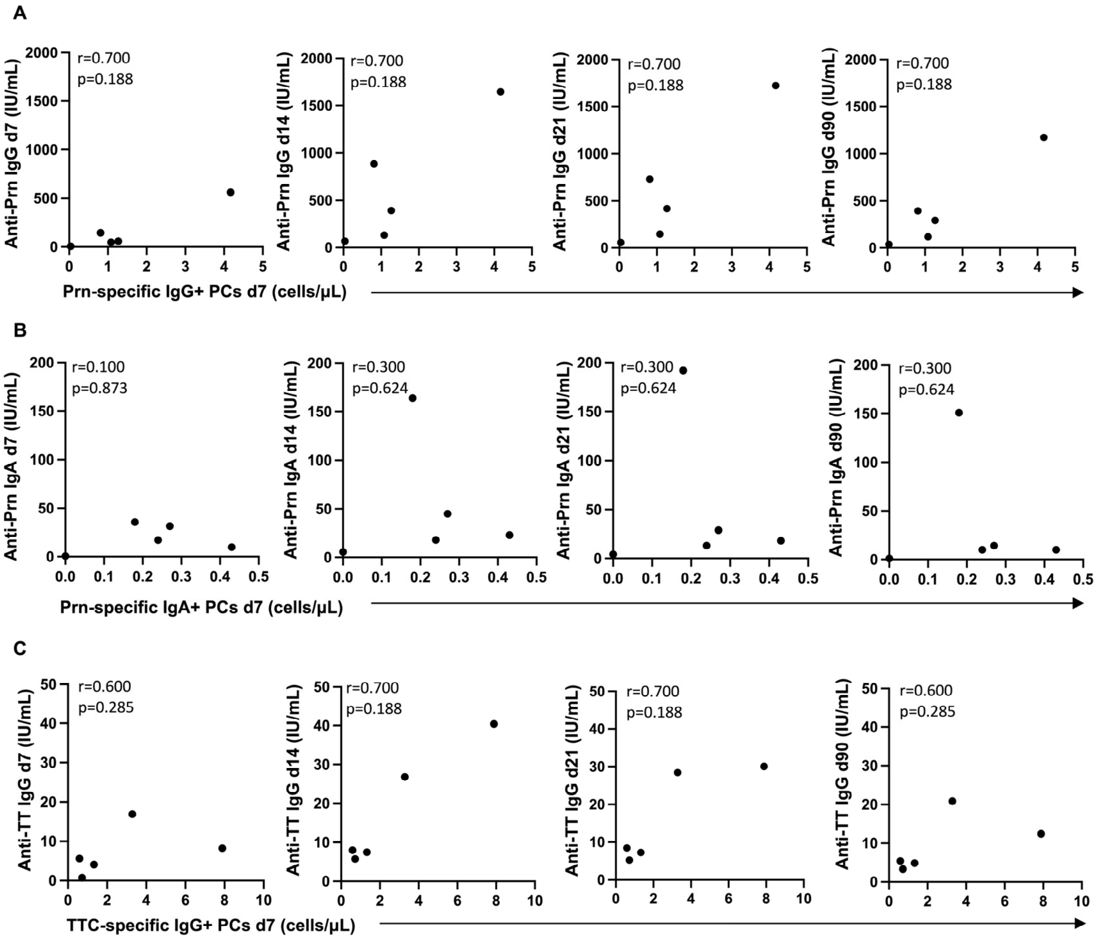

Figure S1. Correlation between vaccine-specific plasma-cell levels at day 7 as measured by flow cytometry and vaccine-specific serum immunoglobulin levels at different time points post-vaccination. Correlation between (A) Prn-specific IgG+ plasma-cell levels at day 7 and anti-Prn IgG levels at day 7, day 14, day 21, and day 90 post-vaccination, (B) Prn-specific IgA+ plasma-cell levels at day 7 and anti-Prn IgA levels at day 7, day 14, day 21, and day 90 post-vaccination, and (C) TTC-specific IgG+ plasma-cell levels at day 7 and anti-TT IgG levels at day 7, day 14, day 21, and day 90 post-vaccination. Each dot represents a single participant. Spearman's Rank Correlation tests were performed to explore the correlation. No significant correlations were observed.

PCs, plasma cells; d, days after vaccination.

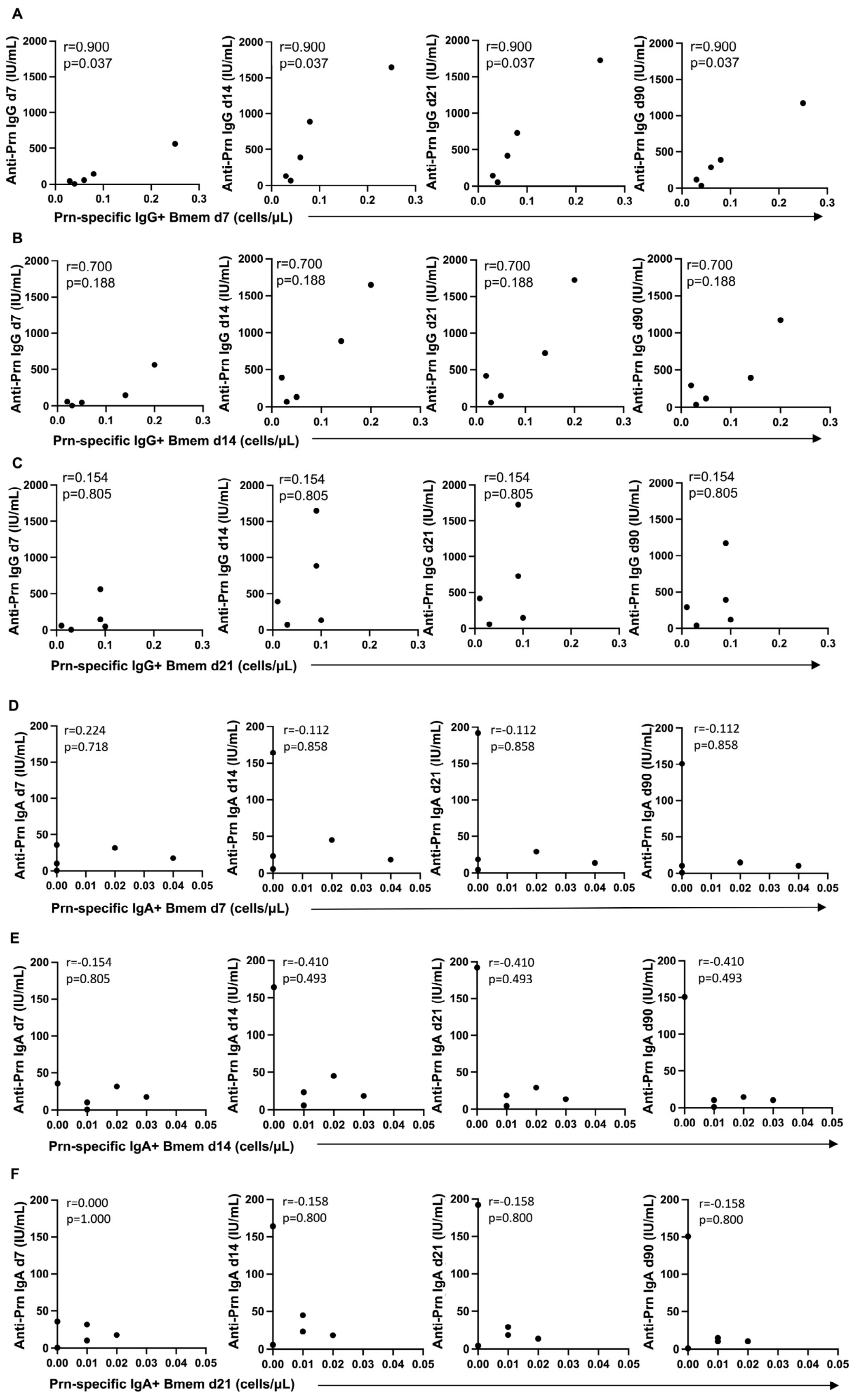

A
r=0.900
p=0.037
Anti-Prn IgG d7 (IU/mL)
Anti-Prn IgG d14 (IU/mL)
Anti-Prn IgG d21 (IU/mL)
Anti-Prn IgG d90 (IU/mL)
Prn-specific IgG+ Bmem d7 (cells/μL)
B
r=0.700
p=0.188
Prn-specific IgG+ Bmem d14 (cells/μL)
C
r=0.154
p=0.805
Prn-specific IgG+ Bmem d21 (cells/μL)
D
r=0.224
p=0.718
r=-0.112
p=0.858
Anti-Prn IgA d7 (IU/mL)
Anti-Prn IgA d14 (IU/mL)
Anti-Prn IgA d21 (IU/mL)
Anti-Prn IgA d90 (IU/mL)
Prn-specific IgA+ Bmem d7 (cells/μL)
E
r=-0.154
p=0.805
r=-0.410
p=0.493
Prn-specific IgA+ Bmem d14 (cells/μL)
F
r=0.000
p=1.000
r=-0.158
p=0.800
Prn-specific IgA+ Bmem d21 (cells/μL)

Figure S2. Correlation between Prn-specific memory B-cell levels at day 7, 14, and 21 as measured by flow cytometry and vaccine-specific serum immunoglobulin levels at different time points post-vaccination. Correlation between (A) Prn-specific IgG+ memory B-cell levels at day 7 and anti-Prn IgG levels at day 7, day 14, day 21, and day 90 post-vaccination, (B) Prn-specific IgG+ memory B-cell levels at day 14 and anti-Prn IgG levels at day 7, day 14, day 21, and day 90 post-vaccination, (C) Prn-specific IgG+ memory B-cell levels at day 21 and anti-Prn IgG levels at day 7, day 14, day 21, and day 90 post-vaccination, (D) Prn-specific IgA+ memory B-cells level at day 7 and anti-Prn IgA levels at day 7, day 14, day 21, and day 90 post-vaccination, (E) Prn-specific IgA+ memory B-cell levels at day 14 and anti-Prn IgA levels at day 7, day 14, day 21, and day 90 post-vaccination, and (F) Prn-specific IgA+ memory B-cell levels at day 21 and anti-Prn IgA levels at day 7, day 14, day 21, and day 90 post-vaccination.

Bmem, memory B cell; d, days after vaccination

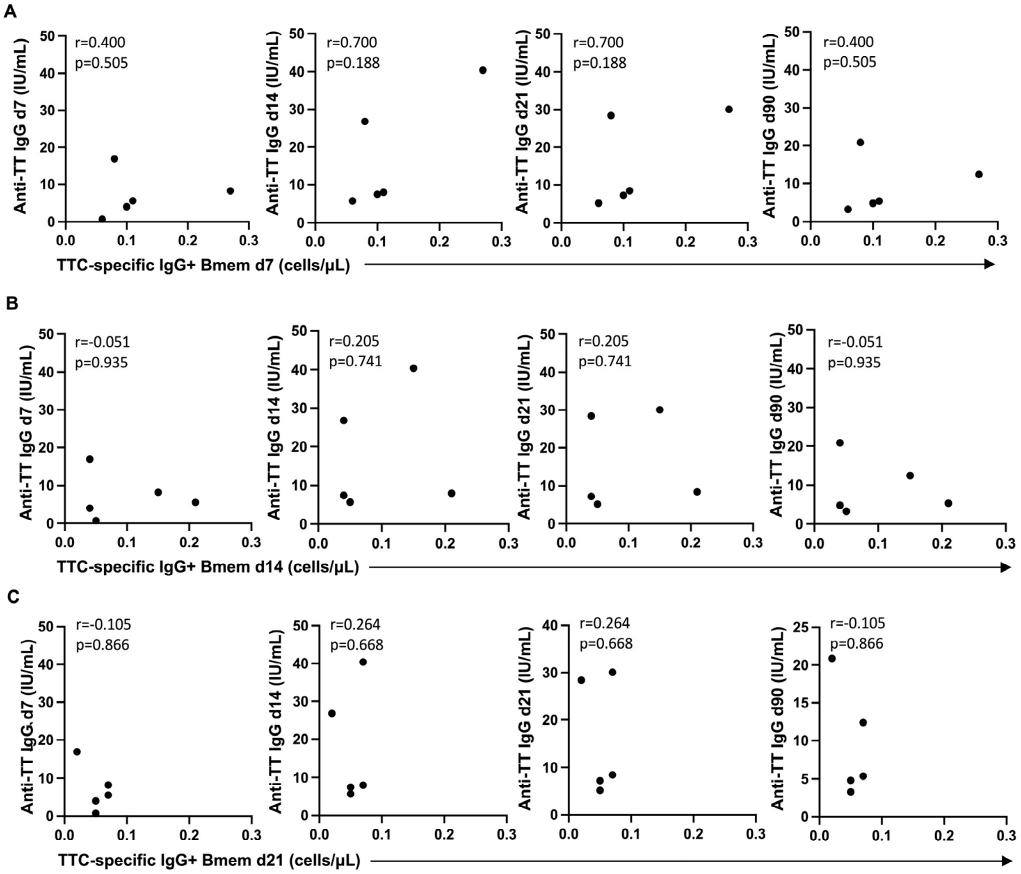

Figure S3. Correlation between TTC-specific memory B-cell levels at day 7, 14, and 21 as measured by flow cytometry and vaccine-specific serum immunoglobulin levels at different time points post-vaccination. Correlation between (A) TTC-specific IgG+ memory B-cell levels at day 7 and anti-TT IgG levels at day 7, day 14, day 21, and day 90 post-vaccination, (B) TTC-specific IgG+ memory B-cell levels at day 14 and anti-TT IgG levels at day 7, day 14, day 21, and day 90 post-vaccination, and (C) TTC-specific IgG+ memory B-cell levels at day 21 and anti-TT IgG levels at day 7, day 14, day 21, and day 90 post-vaccination. Each dot represents a single participant. Spearman's Rank Correlation tests were performed to explore the correlation.

Bmem, memory B cell; d, days after vaccination
